# Energy and Information Beamforming in Airborne Massive MIMO System for Wireless Powered Communications

**DOI:** 10.3390/s18103540

**Published:** 2018-10-19

**Authors:** Yurong Wang, Aijun Liu, Kui Xu, Xiaochen Xia

**Affiliations:** 1 Institute of Communication Engineering, Army Engineering University of PLA, Nanjing 210007, China; wyrbitzj@sina.com (Y.W.); lgdxxukui@ieee.org (K.X.); tjuxxc@sina.com (X.X.); 2 31682 Army of PLA, Lanzhou 730030, China

**Keywords:** airborne massive MIMO, wireless energy transfer, energy and information beamforming, spectral and energy efficiencies

## Abstract

Energy supply and information backhaul are critical problems for wireless sensor networks deployed in remote places with poor infrastructure. To deal with these problems, this paper proposes an airborne massive multiple-input multiple-output (MIMO) system for wireless energy transfer (WET) and information transmission. An air platform (AP) equipped with a two-dimensional rectangular antenna array is employed to broadcast energy and provide wireless access for ground sensors. By exploiting the statistical property of air-terrestrial MIMO channels, the energy and information beamformers are jointly designed to maximize the average received signal-to-interference-plus-noise ratio (SINR), which gives rise to a statistical max-SINR beamforming scheme. The scheme does not rely on the instantaneous channel state information, but still requires large numbers of RF chains at AP. To deal with this problem, a heuristic strongest-path energy and information beamforming scheme is proposed, which can be implemented in the analog-domain with low computational and hardware complexity. The analysis of the relation between the two schemes reveals that, with proper sensor scheduling, the strongest-path beamforming is equivalent to the statistical max-SINR beamforming when the number of AP antennas tends to infinity. Using the asymptotic approximation of average received SINR at AP, the system parameters, including transmit power, number of active antennas of AP and duration of WET phase, are optimized jointly to maximize the system energy efficiency. The simulation results demonstrate that the proposed schemes achieve a good tradeoff between system performance and complexity.

## 1. Introduction

### 1.1. Motivations

In recent years, wireless energy transfer (WET) has been regarded as a promising technology to increase the battery-lifetime of energy-constrained wireless sensor nodes [1,2]. The WET technology allows sensor nodes to harvest energy from surrounding electromagnetic radiation instead of wired energy sources. Many works investigated the WET networks where the traditional ground base station (BS) or access point also acts as an energy station, which can charge the sensor nodes through WET [3]. However, such a configuration is still costly or even infeasible if the sensors are deployed in remote places with poor infrastructure (that may be used for forest fire detection, climate monitoring, etc.). Deploying massive infrastructure in these areas is apparently cost inefficient due to the low population density and hostile environment.

A hybrid air-terrestrial network, where air platforms (APs) are deployed for WET, is a novel solution to deal with this problem. APs are aircraft such as balloons or unmanned aerial vehicles (UAVs) that can broadcast energy and provide wireless access for ground sensors at low cost. Recently, there has emerged a number of applications of AP in wireless networks, but mainly for information transmission [4,5,6,7]. One example is Google Balloon [5], which employs high-altitude balloons to create a hybrid air-terrestrial network with up to an LTE data rate. Another example is “Aquila” proposed by Facebook. In this project, unmanned aerial vehicles are deployed to provide a novel and efficient method of access [6] for ground users. Other than the high-altitude APs, the deployment of low-altitude APs under 1 km for communications has also drawn much attention. In such applications, APs are employed in the high-capacity hot-spot scenario, which is one of the main technical scenarios for the fifth generation (5G) mobile communication systems [7]. In such a scenario, APs play the role of temporary BSs to provide ultra-high data rates for hot-spot users.

Despite its successes in communication applications, AP is still difficult to apply in a WET system directly. One important reason is that, conventionally, AP is equipped with directional antennas that generate several spot beams toward the ground for data transmission [8]. However, when utilized for WET, such an antenna configuration cannot focus the energy to the intended users efficiently [8], and thus gives rise to poor energy efficiency (EE). A promising solution to this problem is to use massive multiple-input multiple-output (MIMO) technology [9] at AP. Through coherent processing for signals of large numbers of antennas, massive MIMO can concentrate the signal power to a compact area around the user efficiently [9,10,11,12,13]. Therefore, a very large power gain can be obtained. This property can be utilized to combat the path loss due to long-range propagation and greatly enlarge the wireless charging distance.

### 1.2. Focus and Contributions

**Figure 1:**
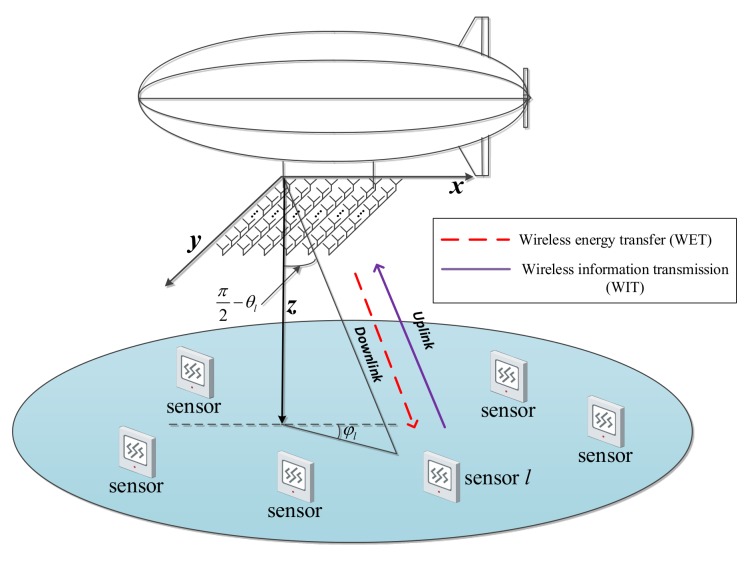
System model.

In this paper, to deal with the energy supply and information backhaul problems for wireless sensor networks in remote places, we propose an airborne massive MIMO system for WET by combining the AP with massive MIMO technology. The system consists of a low-altitude AP equipped with a two-dimensional (2D) rectangular antenna array and a number of sensors on the ground, as shown in Figure 1. The transmission phase is divided into a WET phase during which the AP charges sensors through downlink WET and an uplink wireless information transmission (WIT) phase during which the AP collects data sent by sensors.

In the considered hybrid air-terrestrial network, the biggest challenge is the design of practical energy and information beamforming schemes. Different from the BS of a terrestrial network, AP is usually hardware- and computational complexity-limited due to the consideration of weight, fabricating cost and energy supply [8]. Traditional linear beamforming schemes, such as match filter (MF) beamforming [10] and zero-forcing beamforming [12], require a large number of radio frequency (RF) chains at AP and have high computational complexity. This may make them infeasible when applied on AP. Another challenge lies in the acquisition of instantaneous channel state information (CSI) at the AP. Due to the mobility of AP, the sensor must transmit the uplink pilot sequence frequently, which causes high training overhead and energy consumption. This paper studies the three-dimensional (3D) energy and information beamforming scheme for an airborne massive MIMO system considering the above challenges. The main contributions are as follows.

- By exploiting the statistical property of air-terrestrial MIMO channels, the energy and information beamformers are jointly designed to maximize the average received signal-to-interference-plus-noise ratio (SINR), which gives rise to a statistical max-SINR scheme. Although the scheme does not rely on instantaneous CSI, its implementation still requires large numbers of RF chains at AP. When the number of RF chains is limited, a heuristic strongest-path energy and information beamforming scheme is proposed. The scheme requires only virtual angle of departure (AoD) information of sensors and can be implemented in the analog-domain with low hardware complexity. The analysis of the relation between two schemes reveals that, with proper sensor scheduling, the strongest-path beamforming is equivalent to the statistical max-SINR beamforming when the number of AP’s antennas tends to infinity.
- Based on the asymptotic approximation of average received SINR at AP, the system parameters, including transmit power, number of active antennas of AP and duration of the WET phase, are jointly optimized to maximize the system EE under the proposed strongest-path beamforming scheme.
- Numerical simulations are presented to evaluate the spectral and energy efficiency performances of the proposed beamforming schemes under different system parameters. The results demonstrate the superiority of the proposed schemes in the airborne massive MIMO systems.

### 1.3. Related Literature

The modeling and application of hybrid air-terrestrial networks for access of ground users have drawn much research attention recently. A statistical propagation model has been introduced in [14] to predict the air-to-ground path loss between AP and ground users based on ray tracing simulation. The authors in [15] considered the modeling of small-scale fading for air-to-ground channels, where both line-of-sight (LoS) and non-LoS (NLoS) channel environments were considered. In [4], the authors discussed the basic networking architecture and major design challenges in UAV-aided hybrid air-terrestrial networks. In [16], the authors investigated the 3D beamforming design for a hybrid air-terrestrial network with a large-scale antenna array and analyzed the effect of AoD imperfection on the system performance.

On the other hand, the WET in terrestrial networks has also been extensively investigated for both indoor and outdoor scenarios [17,18,19,20,21,22,23,24,25,26,27]. In [17,18], the authors investigated the outage performance of the indoor WET system and showed that increasing the variance of the log-normal channel would degrade the system performance. In outdoor scenarios, to combat the path loss due to the long charging distance, a number of works considered the utilization of multi-antenna technologies in the WET system. In particular, the benefits and challenges of combining WET with multi-antenna technologies were discussed in [19,20]. The authors in [21] considered the EE optimization in a massive MIMO system with WET and proposed an iterative algorithm to compute the optimal transmit power and duration of the WET phase. The authors in [22] investigated the throughput optimization problem for a WET-enabled massive MIMO system. In [23], an asymptotically optimal downlink power allocation strategy was proposed to maximize the uplink sum rate in a WET-enabled massive MIMO system with linear beamforming. In [24], the 3D massive MIMO was utilized for WET, where the BS applied linear beamforming for energy broadcasting and the users applied power splitting for information detection and energy harvesting. In [25], the energy and information beamforming design for a full-duplex massive MIMO system was investigated. The authors in [26,27] investigated the combined benefits of WET and cooperative relaying systems with large-scale antenna arrays. Due to practical constraints, [19,20,21,22,23,24,25,26,27] have assumed that the energy harvesters (e.g., sensors) are equipped with a single antenna. Recently, several works began to drop this assumption and consider multiple-antenna sensors [28,29,30]. It was shown that, by equipping more antennas at the sensor, the energy harvesting and achievable rate performances can be improved through coherent processing at the sensor side. However, the increase of antennas also incurs problems such as high circuit energy consumption and hardware complexity, which may be unaffordable for sensors. Therefore, in this paper, we assume a single-antenna sensor, and the general case with multiple-antenna sensors will be considered as future work.

### 1.4. Organization and Notations

The rest of the paper is organized as follows. Section 2 presents the system model. Section 3 presents the energy and information beamforming design. Section 4 presents the EE optimization scheme. Section 5 presents the simulation results. Section 6 draws the conclusions.

Notations: The symbol j denotes $\sqrt{-1}$. E(·) denotes the expectation. ⊗ denotes the Kronecker product. ${\left(\cdot \right)}^{*}$, ${\left(\cdot \right)}^{T}$ and ${\left(\cdot \right)}^{H}$ denote the conjugate, transpose and conjugate-transpose of the matrix, respectively. $u_{\max}\left(A\right)$ and $\lambda_{\max}\left(A\right)$ denote the dominant eigenvector and eigenvalue of matrix A. ||·|| denotes the Euclidean norm.

## 2. System Model

### 2.1. Channel Model

Let $h_{l}\in C^{N_{x}N_{y}\times 1}$ denote the channel vector between AP and sensor *l*. Using the air-terrestrial 3D MIMO channel model in [15,31], $h_{l}$ can be expressed as:

(1) $$h_{l}=h_{l}^{\mathrm{LoS}}+h_{l}^{\mathrm{NLoS}}.$$

In (1), $h_{l}^{\mathrm{LoS}}\in C^{N_{x}N_{y}\times 1}$ denotes the deterministic LoS channel component and $h_{l}^{\mathrm{NLoS}}\in C^{N_{x}N_{y}\times 1}$ is the random vector characterizing the non-LoS channel component, which are given by:

(2) $$\begin{array}{c} h_{l}^{\mathrm{LoS}}=\sqrt{\frac{\beta_{l}K_{l}}{K_{l}+1}}b\left(\varphi_{l},\theta_{l}\right)⊗a\left(\varphi_{l},\theta_{l}\right),\\ h_{l}^{\mathrm{NLoS}}=\sqrt{\frac{\beta_{l}}{K_{l}+1}}\int_{\varphi_{l}-\Delta \varphi_{l}}^{\varphi_{l}+\Delta \varphi_{l}}\int_{\theta_{l}-\Delta \theta_{l}}^{\theta_{l}+\Delta \theta_{l}}r_{l}\left(\varphi ,\theta \right)b\left(\varphi ,\theta \right)⊗a\left(\varphi ,\theta \right)d\varphi d\theta , \end{array}$$

with:

(3) $$\begin{array}{cc} a\left(\varphi ,\theta \right)= & \left[1,\exp\left(j\frac{2\pi d_{y}}{\lambda }\cos\theta \cos\varphi \right),\cdots ,\right.{\left.\exp\left(j\frac{2\pi d_{y}\left(N_{y}-1\right)}{\lambda }\cos\theta \cos\varphi \right)\right]}^{T},\\ b\left(\varphi ,\theta \right)= & \left[1,\exp\left(j\frac{2\pi d_{x}}{\lambda }\cos\theta \sin\varphi \right),\cdots ,\right.{\left.\exp\left(j\frac{2\pi d_{x}\left(N_{x}-1\right)}{\lambda }\cos\theta \sin\varphi \right)\right]}^{T}, \end{array}$$

where $\beta_{l}$ and $K_{l}$ denote the large-scale fading and Rician factor, respectively. $\varphi_{l}\in \left[-\pi /2,\pi /2\right]$ and $\theta_{l}\in \left(0,\pi /2\right]$ denote the AoDs of sensor *l* in the horizontal and vertical directions, and $\Delta \varphi_{l}$ and $\Delta \theta_{l}$ denote the corresponding angular spreads. Since AP is much higher than ground obstacles, there is little scatter around it. In this case, the physical signal propagation paths are mainly caused by the scattering process in the vicinity of sensors. This will result in very narrow angular spreads $\Delta \varphi_{l}$ and $\Delta \theta_{l}$ [11]. $d_{x}$ and $d_{y}$ denote the antenna spacings along the *x* axis and *y* axis, respectively. λ denotes the carrier wavelength, and $r_{l}\left(\theta ,\varphi \right)$ denotes the complex response gain associated with the direction (θ,φ). We assume that $r_{l}\left(\theta ,\varphi \right)$ has zero-mean, i.e., $E[r_{l}\left(\theta ,\varphi \right)]=0$, and the response gains for different angles are uncorrelated, i.e.,

(4) $$E\left[r_{l}^{*}\left(\varphi ,\theta \right)r_{l}\left(\varphi^{\prime },\theta^{\prime }\right)\right]=S_{l}\left(\varphi ,\theta \right)\delta \left(\varphi -\varphi^{\prime }\right)\delta \left(\theta -\theta^{\prime }\right),$$

where $S_{l}\left(\varphi ,\theta \right)$ represents the channel power angle spectrum (PAS), which characterizes the channel power distribution in the angular domain. Define $\rho_{x}=\frac{d_{x}}{\lambda }\cos\theta \sin\varphi$ and $\rho_{y}=\frac{d_{y}}{\lambda }\cos\theta \cos\varphi$. Using a similar procedure as that in [16], the channel correlation matrix can be expressed as:

(5) $$\begin{array}{cc} C_{l} & =E\left[h_{l}h_{l}^{H}\right]\\ & \;=\frac{\beta_{l}K_{l}}{K_{l}+1}\left(b\left(\rho_{x,l}\right)b^{H}\left(\rho_{x,l}\right)\right)⊗\left(a\left(\rho_{y,l}\right)a^{H}\left(\rho_{y,l}\right)\right)\\ & \;+\frac{\beta_{l}}{K_{l}+1}\int_{\rho_{x,l}^{\min}}^{\rho_{x,l}^{\max}}\int_{\rho_{y,l}^{\min}}^{\rho_{y,l}^{\max}}S_{l}\left(\rho_{x},\rho_{y}\right)\left(b\left(\rho_{x}\right)b^{H}\left(\rho_{x}\right)\right)⊗\left(a\left(\rho_{y}\right)a^{H}\left(\rho_{y}\right)\right)d\rho_{x}d\rho_{y}\\ & \;\overset{\Delta }{\;=}C_{l}^{\mathrm{LoS}}+C_{l}^{\mathrm{NLoS}} \end{array}$$

with:

(6) $$\begin{array}{c} a\left(\rho_{y}\right)={\left[1,\exp\left(j2\pi \rho_{y}\right),\cdots ,\exp\left(j2\pi \left(N_{y}-1\right)\rho_{y}\right)\right]}^{T},\\ b\left(\rho_{x}\right)={\left[1,\exp\left(j2\pi \rho_{x}\right),\cdots ,\exp\left(j2\pi \left(N_{x}-1\right)\rho_{x}\right)\right]}^{T}, \end{array}$$

where $\rho_{x,l}=\frac{d_{x}}{\lambda }\cos\theta_{l}\sin\varphi_{l}$ and $\rho_{y,l}=\frac{d_{y}}{\lambda }\cos\theta_{l}\cos\varphi_{l}$ denote the virtual AoDs corresponding to the sensors’ physical AoDs in the horizontal and vertical directions. $S_{l}\left(\rho_{x},\rho_{y}\right)$ denotes the PAS with respect to the virtual AoDs $\rho_{x}$ and $\rho_{y}$. The integral boundaries are given by:

(7) $$\begin{array}{c} \;\;\rho_{x,l}^{\max}=\;\;\max_{\theta \in \left[\theta_{l}-\Delta \theta_{l},\theta_{l}+\Delta \theta_{l}\right],\varphi \in \left[\varphi_{l}-\Delta \varphi_{l},\varphi_{l}+\Delta \varphi_{l}\right]}\frac{d_{x}}{\lambda }\cos\left(\theta \right)\sin\left(\varphi \right),\\ \;\;\rho_{x,l}^{\min}=\;\;\min_{\theta \in \left[\theta_{l}-\Delta \theta_{l},\theta_{l}+\Delta \theta_{l}\right],\varphi \in \left[\varphi_{l}-\Delta \varphi_{l},\varphi_{l}+\Delta \varphi_{l}\right]}\frac{d_{x}}{\lambda }\cos\left(\theta \right)\sin\left(\varphi \right),\\ \;\;\rho_{y,l}^{\max}=\;\;\max_{\theta \in \left[\theta_{l}-\Delta \theta_{l},\theta_{l}+\Delta \theta_{l}\right],\varphi \in \left[\varphi_{l}-\Delta \varphi_{l},\varphi_{l}+\Delta \varphi_{l}\right]}\frac{d_{y}}{\lambda }\cos\left(\theta \right)\cos\left(\varphi \right),\\ \;\;\rho_{y,l}^{\min}=\;\;\min_{\theta \in \left[\theta_{l}-\Delta \theta_{l},\theta_{l}+\Delta \theta_{l}\right],\varphi \in \left[\varphi_{l}-\Delta \varphi_{l},\varphi_{l}+\Delta \varphi_{l}\right]}\frac{d_{y}}{\lambda }\cos\left(\theta \right)\cos\left(\varphi \right). \end{array}$$

### 2.2. Energy Transfer and Information Transmission

We adopt frame-based transmission where the length of one frame is denoted by *T*. Each frame consists of a WET phase and a WIT phase.

In the WET phase, the AP charges sensors via downlink energy beamforming. The duration of WET phase is τT with 0<τ<1. According to the law of energy conservation, the harvested energy at sensor *l* can be expressed as [21,22,23] (As in [21,22,23], we have neglected the non-linearity in the energy harvesting process. This is a good assumption for long-range WET, as shown in [32].):

(8) $$\begin{array}{cc} E_{l} & =\eta \tau T\sum_{j=1}^{L}p_{j}{\left|h_{l}^{H}w_{E,j}\right|}^{2}\\ & =\eta \tau Tp_{l}{\left|h_{l}^{H}w_{E,l}\right|}^{2}+\eta \tau T\sum_{j=1,j\neq l}^{L}p_{j}{\left|h_{l}^{H}w_{E,j}\right|}^{2}, \end{array}$$

where $w_{E,j}$ denotes the energy beamformer for sensor *j*. η (0<η<1) denotes the energy conversion efficiency, i.e., the ratio between the harvested energy and received energy. $p_{j}$ denotes the power allocated to the beamformer of sensor *j*, which satisfies $\sum_{j=1}^{L}p_{j}=p_{\mathrm{sum}}$. The first term of the right-hand side indicates the expected energy that can be harvested at sensor *l*, and the second term indicates the energy leakage from the signals sent to other sensors.

Note that the schemes in [19,21,22,23,24,25,26,27,28,29,30] assume that all harvested energy is used for the power amplifier of the sensor during the WIT phase. Different from the previous works, in this paper, we employ a more practical power consumption model for the sensor. In particular, after the WET phase, a part of the harvested energy is used for necessary processing at the sensor to achieve its main task, and the remaining energy is used for uplink transmission during the WIT phase. A similar model has also been applied in [20]. Let $E_{l}^{p}$ denote the processing energy, in the WIT phase of time period (1−τ)T, the transmit power of sensor *l* can be expressed as:

(9) $$\begin{array}{cc} P_{l} & =\frac{E_{l}-E_{l}^{p}}{\left(1-\tau \right)T}\\ & =\frac{\eta \tau }{1-\tau }\sum_{j=1}^{L}p_{j}{\left|h_{l}^{H}w_{E,j}\right|}^{2}-\frac{E_{l}^{p}}{\left(1-\tau \right)T}. \end{array}$$

Equation (9) has assumed that the harvested energy $E_{l}$ is greater than the processing energy $E_{l}^{p}$. Otherwise, $P_{l}$ should be set to zero, and an outage occurs.

To simplify the problem, we assume that the energy leakage, i.e., the second term of (8), contributes little to the total harvested energy. As will be seen later, this assumption is well satisfied under the proposed sensor scheduling scheme. Therefore, the transmit power of sensor *l* in (9) can be approximated as:

(10) $$P_{l}\approx \frac{\eta \tau }{1-\tau }p_{l}{\left|h_{l}^{H}w_{E,l}\right|}^{2}-\frac{E_{l}^{p}}{\left(1-\tau \right)T}.$$

The received signal at AP during the WIT phase is:

(11) $$y=H\Lambda^{1/2}x+n,$$

where $H=\left[h_{1},h_{2},\cdots ,h_{L}\right]$ and $\Lambda =\mathrm{diag}\left\{P_{1},P_{2},\cdots ,P_{L}\right\}$. $x={\left[x_{1},x_{2},\cdots ,x_{L}\right]}^{T}$, and $x_{l}$ denotes the unit-power transmit signal of sensor *l*. n denotes the additive white Gaussian noise (AWGN) vector with distribution $\mathrm{CN}\left(0,\sigma^{2}I_{N_{x}N_{y}}\right)$. To decode the signal of sensor *l*, AP combines the received signal by multiplying the information beamformer $w_{D,l}$, i.e.,

(12) $$y_{l}=\sqrt{P_{l}}w_{D,l}^{H}h_{l}x_{l}+w_{D,l}^{H}\sum_{j=1,j\neq l}^{L}\sqrt{P_{j}}h_{j}x_{j}+w_{D,l}^{H}n.$$

For convenience, we normalize $w_{E,l}$ and $w_{D,l}$ as $∥w_{E,l}∥=∥w_{D,l}∥=1$. The average received SINR of the transmission from sensor *l* to AP can be expressed (since the proposed scheme does not rely on the instantaneous CSI, we consider average received SINR as the performance metric):

(13) $$\begin{array}{cc} E\left[\mathrm{SIN}R_{l}\right] & =E\left[\frac{P_{l}w_{D,l}^{H}h_{l}h_{l}^{H}w_{D,l}}{\sum_{k=1,k\neq l}^{L}P_{k}w_{D,l}^{H}h_{k}h_{k}^{H}w_{D,l}+\sigma^{2}}\right]\\ & \approx \frac{E\left[P_{l}w_{D,l}^{H}h_{l}h_{l}^{H}w_{D,l}\right]}{E\left[\sum_{k=1,k\neq l}^{L}P_{k}w_{D,l}^{H}h_{k}h_{k}^{H}w_{D,l}+\sigma^{2}\right]}. \end{array}$$

The approximation in the second line is based on the Mullens inequality [33], which is widely employed in the literature of massive MIMO. The approximation has been proven tight when the number of antennas (i.e., $N_{x}$ and $N_{y}$) is large [10].

## 3. Energy and Information Beamforming

In this section, we consider the energy and information beamforming design with statistical CSI. When large numbers of RF chains are available at AP, the energy and information beamformers are designed jointly to maximize the average received SINR at AP, which we call the statistical max-SINR beamforming. Although good performance is expected, the scheme causes high computational and hardware complexity when the number of AP’s antennas becomes very large. To deal with this problem, we then propose a heuristic strongest-path energy and information beamforming scheme, which requires only virtual AoD information of sensors and can be implemented in the analog-domain with low hardware complexity. Moreover, with strongest-path beamforming, a simple analytical expression for average received SINR at AP, can be obtained. With this property, an efficient parameter optimization scheme can be developed to further improve the system performance, as will be seen in the next section. The analysis of the relation between two beamforming schemes reveals that, with proper sensor scheduling, the strongest-path beamforming is equivalent to the statistical max-SINR beamforming when the number of AP antennas tends to infinity.

In this section, for information beamforming design, we will assume that the harvested energy is enough, and as a result, the transmit power of sensor given by (10) is positive. When this assumption is not satisfied, the information beamforming problem becomes trivial since the received SINR at AP is always zero.

### 3.1. Statistical Max-SINR Scheme

With only statistical CSI, the beamforming problem is formulated as follows:

(14) $$\begin{array}{c} \max_{w_{E,l},w_{D,l}}E\left[{\mathrm{SINR}}_{l}\right],\\ s.t.\;∥w_{E,l}∥=∥w_{D,l}∥=1. \end{array}$$

Directly solving the above problem is difficult due to the lack of analytical expression of average received SINR. Moreover, the energy beamforming designs for different sensors are coupled through the multi-user interference (MUI) term (i.e., the first term on the denominator of (13)), which makes the problem more challenging.

In this subsection, instead of directly solving the average SINR maximization problem, we first propose a statistical signal-to-leakage-plus-noise ratio (SLNR) criterion to obtain a closed-form solution for the energy beamformer. As its name suggests, the idea is inspired by the conventional SLNR criterion based on instantaneous CSI (referred to as instantaneous SLNR), which has been proven near optimal for the downlink MIMO beamforming problem [34,35]. Then, by exploiting the asymptotic property of the channel covariance matrix, the information beamformer is obtained by solving the problem (14) optimally. Note that in the above strategy, $w_{E,l}$ and $w_{D,l}$ are solved jointly since we have not added any assumption to decouple the problem.

#### 3.1.1. Energy Beamforming Design

Similar to the instantaneous SLNR [34,35], the statistical SLNR is defined as follows:

(15) $${\mathrm{SLNR}}_{l}=\frac{E\left[P_{l}w_{D,l}^{H}h_{l}h_{l}^{H}w_{D,l}\right]}{E\left[P_{l}\sum_{k=1,k\neq l}^{L}w_{D,k}^{H}h_{l}h_{l}^{H}w_{D,k}\right]+\sigma^{2}},$$

where the first term on the denominator indicates the total power of interferences in the WIT phase caused by the transmission of sensor *l*. For convenience, we define $G_{l,k}=w_{D,k}^{H}h_{l}h_{l}^{H}w_{D,k}$ as the combining gain of information beamformer $w_{D,k}$ on the signal of sensor *l*. Substituting the expression of $P_{l}$ given by (10) into (15), the statistical SLNR can be rewritten as:

(16) $$\begin{array}{c} \mathrm{SLN}R_{l}\;\\ =\frac{E\left[\left(\frac{\eta \tau }{1-\tau }p_{l}w_{E,l}^{H}h_{l}h_{l}^{H}w_{E,l}-\frac{E_{l}^{p}}{\left(1-\tau \right)T}\right)G_{l,l}\right]}{E\left[\left(\frac{\eta \tau }{1-\tau }p_{l}w_{E,l}^{H}h_{l}h_{l}^{H}w_{E,l}-\frac{E_{l}^{p}}{\left(1-\tau \right)T}\right)\sum_{k=1,k\neq l}^{L}G_{l,k}\right]+\sigma^{2}}\\ \geq \frac{E\left[\frac{\eta \tau }{1-\tau }p_{l}w_{E,l}^{H}h_{l}h_{l}^{H}w_{E,l}-\frac{E_{l}^{p}}{\left(1-\tau \right)T}\right]E\left[G_{l,l}\right]}{E\left[\left(\frac{\eta \tau }{1-\tau }p_{l}w_{E,l}^{H}h_{l}h_{l}^{H}w_{E,l}-\frac{E_{l}^{p}}{\left(1-\tau \right)T}\right)\sum_{k=1,k\neq l}^{L}G_{l,k}\right]+\sigma^{2}}\\ =\frac{E\left[G_{l,l}\right]w_{E,l}^{H}K_{l}w_{E,l}}{w_{E,l}^{H}\left(K_{l}\sum_{k=1,k\neq l}^{L}E\left[G_{l,k}\right]+\sigma^{2}I_{N_{x}N_{y}}\right)w_{E,l}}\\ \overset{\Delta }{\;=}{\mathrm{SLNR}}_{l}^{\mathrm{LB}}, \end{array}$$

where $K_{l}=\frac{\eta \tau }{1-\tau }p_{l}C_{l}-\frac{E_{l}^{p}}{\left(1-\tau \right)T}I_{N_{x}N_{y}}$. The second line follows from the inequality $E\left[XY\right]-E\left[X\right]E\left[Y\right]=\mathrm{cov}\left(X,Y\right)\geq 0$ when *X* and *Y* are positive.

Based on (16), the energy beamformer is designed to maximize the lower bound of statistical SLNR, i.e.,

(17) $$\max_{w_{E,l}}{\mathrm{SLNR}}_{l}^{\mathrm{LB}},\;s.t.\;∥w_{E,l}∥=1.$$

It is easy to see that (17) is the generalized Rayleigh quotient problem, which can be solved as:

(18) $$w_{E,l}=u_{\max}\left\{{\left(K_{l}\sum_{k=1,k\neq l}^{L}E\left[G_{l,k}\right]+\sigma^{2}I_{N_{x}N_{y}}\right)}^{-1}K_{l}\right\}.$$

Note that the matrices $K_{l}$ and ${\left(K_{l}\sum_{k=1,k\neq l}^{L}E\left[G_{l,k}\right]+\sigma^{2}I_{N_{x}N_{y}}\right)}^{-1}$ have the same eigenvectors with $C_{l}$. Let $\lambda_{i}$ denote the *i*-th largest eigenvalue of $C_{l}$; the *i*-th largest eigenvalue of ${\left(K_{l}\sum_{k=1,k\neq l}^{L}E\left[G_{l,k}\right]+\sigma^{2}I_{N_{x}N_{y}}\right)}^{-1}K_{l}$ can be expressed as:

(19) $${\dot{\lambda }}_{i}=\frac{\frac{\eta \tau }{1-\tau }p_{l}\lambda_{i}-\frac{E_{l}^{p}}{\left(1-\tau \right)T}}{\left(\frac{\eta \tau }{1-\tau }p_{l}\lambda_{i}-\frac{E_{l}^{p}}{\left(1-\tau \right)T}\right)\sum_{k=1,k\neq l}^{L}E\left[G_{l,k}\right]+\sigma^{2}}.$$

Therefore, (18) can be simplified as:

(20) $$w_{E,l}=u_{\max}\left\{C_{l}\right\}.$$

As discussed in [22], the MF energy beamforming aims to maximize the harvested energy at the intended sensor with instantaneous CSI. It is interesting that the energy beamformer (20) under the statistical SLNR criterion gives rise to a similar interpretation in the sense of maximizing the average harvested energy at the intended sensor. Moreover, it is seen that the solution of the energy beamformer is decoupled with the information beamformer. This makes the closed-form solution for $w_{D,l}$ possible.

#### 3.1.2. Information Beamforming Design

By substituting (20) into (14), the information beamforming problem can be expressed as follows:

(21) $$\begin{array}{c} \max_{w_{D,l}}\frac{w_{D,l}^{H}E\left[P_{l}h_{l}h_{l}^{H}\right]w_{D,l}}{w_{D,l}^{H}\left(\sum_{k=1,k\neq l}^{L}E\left[P_{k}h_{k}h_{k}^{H}\right]+\sigma^{2}\right)w_{D,l}},\\ s.t.∥w_{D,l}∥=1. \end{array}$$

Again, (21) is in the form of generalized Rayleigh quotient problem, which can be solved as:

(22) $$\begin{array}{cc} w_{D,l} & =u_{\max}\left\{{\left(\sum_{k=1,k\neq l}^{L}E\left[P_{k}h_{k}h_{k}^{H}\right]+\sigma^{2}I_{N_{x}N_{y}}\right)}^{-1}E\left[P_{l}h_{l}h_{l}^{H}\right]\right\}. \end{array}$$

Since the transmit power $P_{l}$ is correlated with the channel $h_{l}$, there is no closed-form expressions for the expectations in (21). Therefore, the information beamforming based on (22) requires numerical evaluation with high computational complexity.

To overcome this challenge, below, we propose a closed-form solution for $w_{D,l}$ based on the asymptotic property of the sensor’s transmit power $P_{l}$.

**Lemma** **1.** *When the number of AP antennas tends to infinity, i.e., $\left\{N_{x},N_{y}\right\}\to \infty$, the transmit power of sensor l converges to:*

(23) $$P_{l}\approx \frac{\eta \tau }{1-\tau }p_{l}\frac{\beta_{l}K_{l}}{K_{l}+1}N_{x}N_{y}-\frac{E_{l}^{p}}{\left(1-\tau \right)T}.$$

**Proof.**  See Appendix A. □

Inserting (23) into (22), we get a closed-form solution for $w_{D,l}$ as follows:

(24) $$\begin{array}{cc} w_{D,l} & =u_{\max}\left\{\left(\sum_{k=1,k\neq l}^{L}\left(\frac{\eta \tau }{1-\tau }p_{k}\frac{\beta_{k}K_{k}}{K_{k}+1}N_{x}N_{y}\right.\right.{\left.\left.-\frac{E_{k}^{p}}{\left(1-\tau \right)T}\right)C_{k}+\sigma^{2}I_{N_{x}N_{y}}\right)}^{-1}C_{l}\right\}. \end{array}$$

Even if closed-form expressions are available, the computation of beamformers in (20) and (24) has complexity $O\left(N_{x}^{3}N_{y}^{3}\right)$, which becomes challenging when the number of AP antennas grows large. Moreover, to implement the beamforming scheme, the required number of RF chains at AP is equal to $N_{x}N_{y}$. Since AP is hardware complexity limited, it is very important to reduce the number of required RF chains at AP for energy and information beamforming. This will be considered in the next subsection.

### 3.2. Strongest-Path Beamforming Scheme

In this subsection, we present a two-step heuristic approach to obtain a low-complexity beamforming scheme. In the first step, we design the energy and information beamformers jointly to maximize the average power of useful signal (i.e., the numerator of (13)), without considering the power of MUI (i.e., the first term on the denominator of (13)). In the second step, we propose an MUI-aware sensor scheduling scheme to mitigate the MUI under the beamforming scheme obtained in the first step.

#### 3.2.1. Average Useful Signal Power Maximization

According to (13), the average power of useful signal can be expressed as:

(25) $$Q_{l}^{U}=E\left[P_{l}w_{D,l}^{H}h_{l}h_{l}^{H}w_{D,l}\right].$$

Directly maximizing $Q_{l}^{U}$ with respect to $w_{E,l}$ and $w_{D,l}$ is still difficult. By using the inequality $E\left[XY\right]-E\left[X\right]E\left[Y\right]=\mathrm{cov}\left(X,Y\right)\geq 0$ for positive $\left\{X,Y\right\}$, a lower bound on $Q_{l}^{U}$ can be obtained as:

(26) $$\begin{array}{cc} Q_{l}^{U} & \geq E\left[P_{l}\right]E\left[w_{D,l}^{H}h_{l}h_{l}^{H}w_{D,l}\right]\\ & =w_{E,l}^{H}K_{l}w_{E,l}w_{D,l}^{H}C_{l}w_{D,l}\overset{\Delta }{\;=}Q_{l}^{U,\mathrm{LB}}. \end{array}$$

Since $K_{l}$ has the same eigenvectors as $C_{l}$, designing the energy and information beamformers to maximize the lower bound gives rise to the solution as follows:

(27) $$w_{E,l}=w_{D,l}=u_{\max}\left\{C_{l}\right\}.$$

By exploiting the asymptotic property of the channel covariance matrix, we can further approximate the energy and information beamformers using the virtual AoD information of the sensor. This is stated in the following theorem.

**Theorem** **1.** *When the number of AP antennas tends to infinity, i.e., $\left\{N_{x},N_{y}\right\}\to \infty$, the energy and information beamformers converge to:*

(28) $$w_{E,l}=w_{D,l}=\frac{1}{\sqrt{N_{x}N_{y}}}b\left(\rho_{x,l}\right)⊗a\left(\rho_{y,l}\right),$$

*and the lower bound of average useful signal power can be expressed as:*

(29) $$\begin{array}{cc} Q_{l}^{U,\mathrm{LB}} & =\left\{\frac{\eta \tau }{1-\tau }p_{l}\left(\frac{\beta_{l}K_{l}}{K_{l}+1}N_{x}N_{y}+\frac{\beta_{l}}{K_{l}+1}S_{l}\left(\rho_{x,l},\rho_{y,l}\right)\right)\right.\\ & \left.-\frac{E_{l}^{p}}{\left(1-\tau \right)T}\right\}\left(\frac{\beta_{l}K_{l}}{K_{l}+1}N_{x}N_{y}+\frac{\beta_{l}}{K_{l}+1}S_{l}\left(\rho_{x,l},\rho_{y,l}\right)\right). \end{array}$$

**Proof.**  This can be readily proven using the asymptotic expressions for the dominant eigenvector and eigenvalue of $C_{l}$, presented in (A6) of Appendix A. □

From Theorem 1, it is seen that the beamformers are designed to match the LoS path of the channel, i.e., to ensure that the largest beamforming gain is achieved at the LoS path. Moreover, for the commonly-used models, the PAS of NLoS channel $S_{l}\left(\rho_{x},\rho_{y}\right)$ achieves the maximum at $\left\{\rho_{x},\rho_{y}\right\}=\left\{\rho_{x,l},\rho_{y,l}\right\}$ [15,36], i.e., the virtual AoDs corresponding to the sensors’ physical AoDs in horizontal and vertical directions. As a result, the proposed beamformers also match the strongest NLoS path in the angular domain. Therefore, this scheme is named the strongest-path energy and information beamforming. On the other hand, it is seen that both the energy and information beamformers have a constant envelope, which can be realized using phase shifting networks in the analog-domain. The total number of required RF chains at AP can be reduced to *L*, which is equal to the number of sensors [37].

Another important observation from Lemma 1 is that, if we neglect the processing energy $E_{l}^{p}$, the average useful signal power decays with the squared path loss. Thus, a low altitude of AP is preferred to improve the efficiencies of WET and WIT of the single sensor. However, with the decrease of altitude, the number of covered sensors by AP is reduced as well, which may degrade the performance of the whole hybrid air-terrestrial network. Therefore, the planning of AP’s altitude is an interesting and important research topic. If the prior of sensor density is available, a raw idea to solve this problem is presented as follows. Note that for the given altitude of AP, the uplink achievable rate region of the sensor can be predicted based on (29) or the analytical SINR expression presented in Section 4. Moreover, the performance metric of the network, e.g., system spectral efficiency (SE), can be estimated using the sensor density information. With these in the hand, the altitude optimization can be formulated as a network performance maximization problem subject to the sensor quality of service (QoS) constraint, e.g., a threshold on the uplink achievable rate.

#### 3.2.2. MUI Mitigation

According to (13) and Lemma 1, the power of MUI can be expressed asymptotically in the large $\left\{N_{x},N_{y}\right\}$ limit as:

(30) $$\begin{array}{cc} Q_{l}^{\mathrm{MUI}} & =\sum_{k=1,k\neq l}^{L}E\left[P_{k}w_{D,l}^{H}h_{k}h_{k}^{H}w_{D,l}\right]\\ & =\sum_{k=1,k\neq l}^{L}\left(\frac{\eta \tau }{1-\tau }p_{l}\frac{\beta_{l}K_{l}}{K_{l}+1}N_{x}N_{y}-\frac{E_{l}^{p}}{\left(1-\tau \right)T}\right)E\left[w_{D,l}^{H}h_{k}h_{k}^{H}w_{D,l}\right]. \end{array}$$

By exploiting the channel covariance matrix (5) and the expression of $w_{D,l}$ given by Theorem 1, $Q_{l}^{\mathrm{MUI}}$ can be rewritten as:

(31) $$\begin{array}{cc} Q_{l}^{\mathrm{MUI}} & =\sum_{k=1,k\neq l}^{L}\left(\frac{\eta \tau }{1-\tau }p_{k}\frac{\beta_{k}K_{k}}{K_{k}+1}N_{x}N_{y}-\frac{E_{k}^{p}}{\left(1-\tau \right)T}\right)\\ & \times \frac{\beta_{k}}{K_{k}+1}\left(\frac{1}{N_{x}N_{y}}\prod_{i\in \left\{x,y\right\}}\frac{\sin^{2}\left(N_{x}\pi \left(\rho_{i,l}-\rho_{i,k}\right)\right)}{\sin^{2}\left(\pi \left(\rho_{i,l}-\rho_{i,k}\right)\right)}+\frac{1}{N_{x}N_{y}}\right.\\ & \left.\times \int_{\rho_{c_{k},x}^{\min}}^{\rho_{c_{k},x}^{\max}}\int_{\rho_{c_{k},y}^{\min}}^{\rho_{c_{k},y}^{\max}}S_{k}\left(\rho_{x},\rho_{y}\right)\;\;\;\prod_{i\in \left\{x,y\right\}}\frac{\sin^{2}\left(N_{x}\pi \left(\rho_{i,l}-\rho_{i}\right)\right)}{\sin^{2}\left(\pi \left(\rho_{i,l}-\rho_{i}\right)\right)}d\rho_{x}d\rho_{y}\right). \end{array}$$

A short derivation of (31) is presented in Appendix B.

Before proposing the MUI-aware sensor scheduling scheme, we first present the following lemma, which determines the scaling behavior of MUI with respect to $\left\{N_{x},N_{y}\right\}$.

**Lemma** **2.** *Consider the following two conditions on the virtual AoDs of sensors: (a) $\left[\rho_{x,l}^{\min},\rho_{x,l}^{\max}\right]\cap \left[\rho_{x,k}^{\min},\rho_{x,k}^{\max}\right]=\emptyset$, ∀l≠k, which indicates that the virtual AoD regions along the x-direction of any two sensors are non-overlapping. (b) $\left[\rho_{y,l}^{\min},\rho_{y,l}^{\max}\right]\cap \left[\rho_{y,k}^{\min},\rho_{y,k}^{\max}\right]=\emptyset$, ∀l≠k, which indicates that the virtual AoD regions along the y-direction of any two sensors are non-overlapping. In the large $\left\{N_{x},N_{y}\right\}$ limit,*

- *if the virtual AoDs of sensors satisfy only one of Conditions (a) and (b), the interference-to-useful signal ratio scales at most with $O\left(N_{x}^{-2}\right)$ (if Condition (a) holds) or $O\left(N_{y}^{-2}\right)$ (if Condition (b) holds).*
- *if Conditions (a) and (b) are satisfied simultaneously, the power of MUI scales with $O\left(N_{x}^{-1}N_{y}^{-1}\right)$.*

**Proof.**  See Appendix C. □

Lemma 2 indicates that: (1) in the low received SNR scenario, the effect of MUI can be efficiently mitigated if the sensors are scheduled to make Condition (a) or Condition (b) satisfied; (2) whereas in the high received SNR scenario, a good sensor scheduling scheme should make Conditions (a) and (b) hold simultaneously.

Based on Lemma 2 and the above discussion, we can build an MUI-aware sensor scheduling scheme as follows.

**Figure 2:**
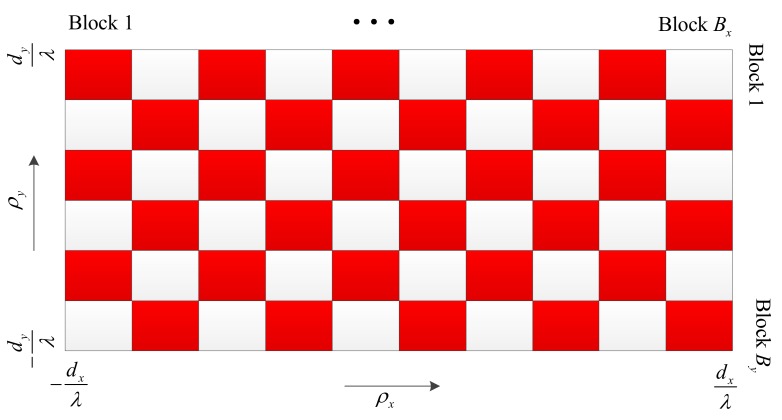
Illustration of the MUI-aware sensor scheduling scheme.

- In the first step, we divide the whole region of virtual AoD along the *x*-direction into $B_{x}$ disjoint blocks, and the length of each block is much larger than $\max_{l}\left\{\rho_{x,l}^{\max}-\rho_{x,l}^{\min}\right\}$. Similarly, the whole region of virtual AoD along the *y*-direction is divided into the $B_{y}$ disjoint blocks, and the length of each block is much larger than $\max_{l}\left\{\rho_{y,l}^{\max}-\rho_{y,l}^{\min}\right\}$, as shown in Figure 2.
- In the second step, the sensors whose virtual AoDs $\left\{\rho_{x,l},\rho_{y,l}\right\}$ lie in the same small rectangular region in Figure 2 are gathered into one group. In this way, we will have at most $B_{x}B_{y}$ sensor groups.
- In the third step, for the low received SNR scenario, we pick one sensor from each group to serve a particular time-frequency resource. Thus, the maximum number of simultaneously served sensors is equal to $B_{x}B_{y}$. In the high received SINR scenario, the scheduled sensors are selected in the same way, but further divided into two clusters, as shown in Figure 2, where the sensors from red regions are assigned to Cluster 1 and those from white regions are assigned to Cluster 2. Each cluster is served on different time-frequency resources. Thus, the maximum number of simultaneously served sensors is equal to $\frac{1}{2}B_{x}B_{y}$. To ensure the fairness between all sensors, the remaining sensors in each group can be served on the other time-frequency resources using the same scheduling procedure.

Since in the first step, we have made the length of each block much larger than the spread of virtual AoD of each sensor, i.e., $\rho_{x,l}^{\max}-\rho_{x,l}^{\min}$ and $\rho_{y,l}^{\max}-\rho_{y,l}^{\min}$, the proposed scheme can make Condition (a) and/or (b) satisfied approximately. In this case, the power of MUI is greatly mitigated. From the first and third steps, $B_{x}$ and $B_{y}$ can be viewed as the parameters that give a tradeoff between the allowed residual MUI and the number of simultaneously served sensors. Thus, considering the constraint in the first step, $B_{x}$ and $B_{y}$ should be designed to maximize some performance metric (e.g., the system SE). In practical application, since the feasible sets $B_{x}$ and $B_{y}$ are discrete and finite, this task can be completed by simple off-line searching algorithms based on the analytical results in Theorem 1 and (31).

**Remark** **1.** *Note that both the strongest-path beamforming and sensor scheduling require the knowledge of virtual AoDs of sensors. By using the class of estimation methods based on discrete Fourier transform with zero padding [38], the virtual AoDs can be estimated at AP from the pilots transmitted by sensors with a certain quantification error.*

**Remark** **2.** *If the remaining battery power of a sensor is not enough to transmit the pilot signal, AP cannot obtain its AoD estimation. In this case, this sensor becomes a “dead sensor”, which can no longer be severed. To deal with this problem, the common signal of AP can be exploited. Usually, AP will broadcast periodically some common signals within its coverage region for the purpose of synchronization, access control, etc. The sensor with low battery power can harvest energy from these common signals until it has enough battery power to access the network actively. Some methods of designing an omnidirectionally common signal in a massive MIMO system can be found in [39].*

### 3.3. Relation between Two Beamforming Schemes

According to (20) and (27) and Theorem 1, we can see that the energy beamformer of the statistical max-SINR scheme converges to that of the strongest-path beamforming in the large $\left\{N_{x},N_{y}\right\}$ limit. Moreover, if Conditions (a) and/or (b) are satisfied after the MUI-aware sensor scheduling, we can neglect the term incurred by MUI, i.e., $\sum_{k=1,k\neq l}^{L}\left(\frac{\eta \tau }{1-\tau }p_{k}\frac{\beta_{k}K_{k}}{K_{k}+1}N_{x}N_{y}\right.\left.-\frac{E_{k}^{p}}{\left(1-\tau \right)T}\right)C_{k}$, in the information beamformer given by (24), and rewrite (24) as:

(32) $$\begin{array}{cc} w_{D,l} & =u_{\max}\left\{C_{l}\right\}\overset{N_{x},N_{y}\to \infty }{=}\frac{1}{\sqrt{N_{x}N_{y}}}b\left(\rho_{x,l}\right)⊗a\left(\rho_{y,l}\right). \end{array}$$

Therefore, we can conclude that, under the MUI-aware sensor scheduling, the strongest-path beamforming is equivalent to the statistical max-SINR beamforming when the number of AP antennas tends to infinity.

## 4. Energy Efficiency Optimization

In this section, the transmit power, number of active antennas of AP and duration of the WET phase are jointly optimized to maximize the system EE. It is assumed that the low-complexity strongest-path beamforming scheme is employed at AP. Based on the theoretical analysis in Section 3.2, an approximate expression of average received SINR at AP can be obtained as:

(33) $$E\left[{\mathrm{SINR}}_{l}\right]\approx \frac{Q_{l}^{U,\mathrm{LB}}}{Q_{l}^{\mathrm{MUI}}+\sigma^{2}},$$

where the expressions of $Q_{l}^{U,\mathrm{LB}}$ and $Q_{l}^{\mathrm{MUI}}$ are given by (29) and (31), respectively.

We assume that the MUI is effectively mitigated after the proposed MUI-aware sensor scheduling scheme, and as a result, the system is noise-limited (if the effect MUI is not negligible, by exploiting the analytical expression in (31), the EE optimization problem can be solved efficiently using the geometric programming based procedure developed in [40]). Thus, by substituting (29) into (33) and neglecting the low-order terms, the average received SINR can be further simplified as:

(34) $$E\left[{\mathrm{SINR}}_{l}\right]\approx \frac{\tau N_{x}^{2}N_{y}^{2}}{1-\tau }\alpha_{l}p_{l}-\frac{N_{x}N_{y}}{1-\tau }\gamma_{l},$$

where $\alpha_{l}=\frac{\eta }{\sigma^{2}}{\left(\frac{\beta_{l}K_{l}}{K_{l}+1}\right)}^{2}$ and $\gamma_{l}=\frac{E_{p}}{\sigma^{2}T}\frac{\beta_{l}K_{l}}{K_{l}+1}$.

The EE is defined as the SE divided by the AP’s total energy consumption, that is:

(35) $$\mathrm{EE}=\frac{\mathrm{SE}}{\tau T\sum_{l=1}^{L}p_{l}+TP_{c}},$$

where the SE is defined as the sum of all sensors’ uplink achievable rates, that is:

(36) $$\mathrm{SE}=\left(1-\tau \right)\sum_{l=1}^{L}\log\left(1+\frac{\tau N_{x}^{2}N_{y}^{2}}{1-\tau }\alpha_{l}p_{l}-\frac{N_{x}N_{y}}{1-\tau }\gamma_{l}\right),$$

and $P_{c}$ denotes the circuit power consumption of AP. According to [21,41], it is reasonable to assume that the circuit power consumption increases linearly with the number of the AP’s active antennas. Thus, $P_{c}$ can be expressed as:

(37) $$P_{c}={\tilde{N}}_{x}{\tilde{N}}_{y}p_{\mathrm{ant}},$$

where ${\tilde{N}}_{x}$ and ${\tilde{N}}_{y}$ denote the numbers of active antennas along the *x* axis and *y* axis, respectively, which satisfy ${\tilde{N}}_{x}\leq N_{x}$ and ${\tilde{N}}_{y}\leq N_{y}$. $p_{\mathrm{ant}}$ denotes the power consumption introduced by each active antenna.

We consider the EE optimization problem with the per sensor achievable rate constraint, which is formulated as:

(38) $$\begin{array}{c} \max_{\tau ,{\left\{p_{l}\right\}}_{l=1}^{L},{\tilde{N}}_{x},{\tilde{N}}_{y}}\mathrm{EE},\\ s.t\left\{\begin{array}{c} C_{1}:\left(1-\tau \right)\log\left(1+\frac{\tau N_{x}^{2}N_{y}^{2}}{1-\tau }\alpha_{l}p_{l}-\frac{N_{x}N_{y}}{1-\tau }\gamma_{l}\right)\geq r_{T},\\ l=1,\cdots ,L,\\ C_{2}:\sum_{l=1}^{L}p_{l}\leq p_{\max},p_{l}\geq 0,l=1,\cdots ,L,\\ C_{3}:{\tilde{N}}_{x}\leq N_{x},{\tilde{N}}_{y}\leq N_{y},\;\mathrm{and}\;{\tilde{N}}_{x},{\tilde{N}}_{y}\in N^{+},\\ C_{4}:\tau \in \left(0,1\right). \end{array}\right. \end{array}$$

where $r_{T}$ denotes the minimum data rate requirement of each sensor. $p_{\max}$ denotes the maximum transmit power constraint of AP. Note that the constraint $C_{1}$ implicitly enforces the harvested energy to be greater than the processing energy. Thus, no additional constraint is needed. Due to the tremendous gain of a large-scale antenna array in a massive MIMO system, it is reasonable to assume that the uplink received SINR given by (34) is much greater than one. Thus, at a good operating point, the increase of $N_{x}$, $N_{y}$ and $p_{l}$ will result in the decrease of EE since the achievable rate grows logarithmically with $N_{x}$, $N_{y}$ and $p_{l}$. In this case, the constraint $C_{1}$ will satisfy with equality at optimal $N_{x}$, $N_{y}$ and $p_{l}$. Therefore, by substituting (35) and (37) into (38) and exploiting the definition of SE, the EE optimization problem can be rewritten as:

(39) $$\begin{array}{c} \min_{\tau ,{\left\{p_{l}\right\}}_{l=1}^{L}{\tilde{N}}_{x},{\tilde{N}}_{y}}\tau \sum_{l=1}^{L}p_{l}+{\tilde{N}}_{x}{\tilde{N}}_{y}p_{\mathrm{ant}},\\ s.t.\left\{\begin{array}{c} \left(1-\tau \right)\log\left(1+\frac{\tau N_{x}^{2}N_{y}^{2}}{1-\tau }\alpha_{l}p_{l}-\frac{N_{x}N_{y}}{1-\tau }\gamma_{l}\right)=r_{T},\\ l=1,\cdots ,L,\\ C_{2}∼C_{4}. \end{array}\right. \end{array}$$

By rearranging the constraint $C_{1}$, we can obtain:

(40) $$p_{l}=\frac{1-\tau }{\tau \alpha_{l}{\tilde{N}}_{x}^{2}{\tilde{N}}_{y}^{2}}\left\{\exp\left(\frac{r_{T}}{1-\tau }\right)-1+\frac{{\tilde{N}}_{x}{\tilde{N}}_{y}}{1-\tau }\gamma_{l}\right\}.$$

With this equality, we can rewrite the problem (39) as:

(41) $$\begin{array}{c} \min_{\tau ,{\tilde{N}}_{x},{\tilde{N}}_{y}}\sum_{l=1}^{L}\frac{1-\tau }{\alpha_{l}}\left\{\exp\left(\frac{r_{T}}{1-\tau }\right)-1\right\}\frac{1}{{\tilde{N}}_{x}^{2}{\tilde{N}}_{y}^{2}}+\sum_{l=1}^{L}\frac{\gamma_{l}}{\alpha_{l}}\frac{1}{{\tilde{N}}_{x}{\tilde{N}}_{y}}+{\tilde{N}}_{x}{\tilde{N}}_{y}p_{\mathrm{ant}},\\ s.t.\left\{\begin{array}{c} \sum_{l=1}^{L}\frac{1-\tau }{\tau \alpha_{l}{\tilde{N}}_{x}^{2}{\tilde{N}}_{y}^{2}}\left\{\exp\left(\frac{r_{T}}{1-\tau }\right)-1+\frac{{\tilde{N}}_{x}{\tilde{N}}_{y}}{1-\tau }\gamma_{l}\right\}\leq p_{\max},\\ C_{3},C_{4}. \end{array}\right. \end{array}$$

With a few algebraic manipulations, we can see that the inequality constraint in (41) gives rise to a lower bound on ${\tilde{N}}_{x}{\tilde{N}}_{y}$, that is:

(42) $$\begin{array}{cc} {\tilde{N}}_{x}{\tilde{N}}_{y} & \geq \frac{\sum_{l=1}^{L}\frac{\gamma_{l}}{\tau \alpha_{l}}\;\;+\;\;\sqrt{{\left(\sum_{l=1}^{L}\frac{\gamma_{l}}{\tau \alpha_{l}}\right)}^{2}\;\;-\;4p_{\max}\sum_{l=1}^{L}\frac{1-\tau }{\tau \alpha_{l}}\left\{\exp\left(\frac{r_{T}}{1-\tau }\right)\;\;-\;\;1\right\}}}{2p_{\max}}\\ & \overset{\Delta }{\;=}N_{\mathrm{LB}}. \end{array}$$

Moreover, setting the first-order derivative of the target function with respect to ${\tilde{N}}_{x}{\tilde{N}}_{y}$ to zero, we can obtain:

(43) $$-\frac{2\sum_{l=1}^{L}\frac{1-\tau }{\alpha_{l}}\left\{\exp\left(\frac{r_{T}}{1-\tau }\right)-1\right\}}{{\tilde{N}}_{x}^{3}{\tilde{N}}_{y}^{3}}-\sum_{l=1}^{L}\frac{\gamma_{l}}{\alpha_{l}}\frac{1}{{\tilde{N}}_{x}^{2}{\tilde{N}}_{y}^{2}}+p_{\mathrm{ant}}=0.$$

Since (43) is a monotonically increasing function of ${\tilde{N}}_{x}{\tilde{N}}_{y}$, it has a single solution (denoted as ${\tilde{N}}_{x}{\tilde{N}}_{y}=N^{†}$) when ${\tilde{N}}_{x}{\tilde{N}}_{y}>0$. Therefore, the target function achieves its minimum at ${\tilde{N}}_{x}{\tilde{N}}_{y}=N^{†}$. Note that $N^{†}$ can be simply obtained using the method of bisection. Considering the lower bound in (42), if we discard the integer constraints on ${\tilde{N}}_{x}$ and ${\tilde{N}}_{y}$, the optimal ${\tilde{N}}_{x}$ and ${\tilde{N}}_{y}$ satisfy:

(44) $${\tilde{N}}_{x}{\tilde{N}}_{y}=\max\left\{N_{\mathrm{LB}},N^{†}\right\}.$$

When the right-hand side of (44) is greater than $N_{x}N_{y}$, the problem is infeasible. As long as the problem is feasible, ${\tilde{N}}_{x}$ and ${\tilde{N}}_{y}$ can be selected as arbitrary positive integers, which can make (44) satisfied.

After determining the number of active antennas, the optimal transmit power of AP can be derived directly using (40). Finally, the optimal τ can be obtained by one-dimensional search over the interval (0,1).

From the above solutions, we can obtain two important observations on the design of system parameters.

- First, for the above EE and widely-investigated SE optimization problems [12], we note that the optimal transmit powers derived have distinct scaling behaviors with respect to the number of the AP’s (active) antennas. According to (40), the optimal transmit power that maximizes the EE reduces approximately with ${\tilde{N}}_{x}^{2}{\tilde{N}}_{y}^{2}$ if the processing energy $E_{p}$ is negligible and reduces with ${\tilde{N}}_{x}{\tilde{N}}_{y}$ if the processing energy is significant. In contrast, in SE optimization, the optimal transmit power is independent of the number of AP antennas [12].
- In EE optimization, the optimal number of active antennas, i.e., ${\tilde{N}}_{x}{\tilde{N}}_{y}$, is a non-increasing function of transmit power constraint $p_{\max}$ and the circuit power consumption $p_{\mathrm{ant}}$. In particular, when the circuit power consumption is dominant, (44) reduces to ${\tilde{N}}_{x}{\tilde{N}}_{y}=N_{\mathrm{LB}}$, which becomes independent of $p_{\mathrm{ant}}$. In this case, by substituting (40) into the constraint $C_{2}$ of (38), we can see that the sum transmit power constraint of AP holds with equality. This means that, to control the total circuit power consumption, AP should use its maximum transmit power to reduce the number of active antennas.

## 5. Simulation Results and Discussion

**Figure 3:**
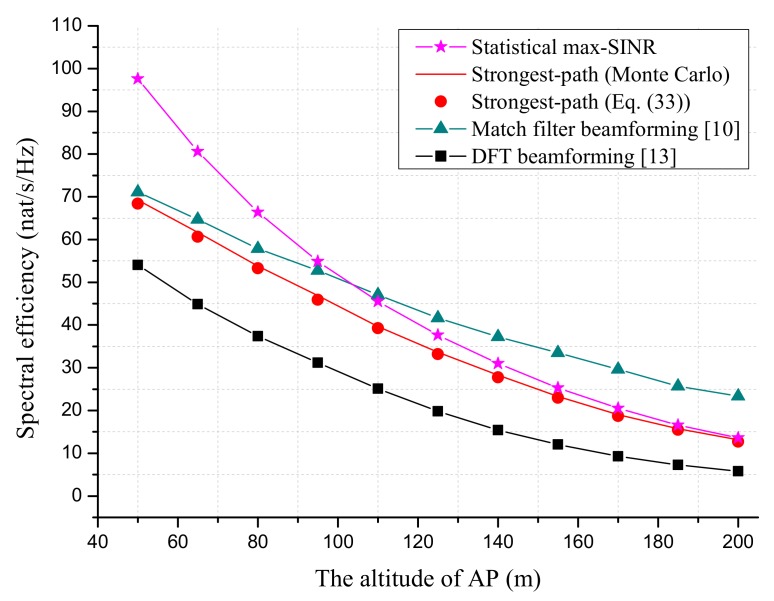
The SE performance as a function of AP’s altitude. The number of AP antennas is $N_{x}=N_{y}=25$. The total transmit power of AP is set to $\sum_{l=1}^{L}p_{l}=40$ dBm. The processing energy of sensor is set to $E_{l}^{p}=10^{-10}\;J$.

This section evaluates the performance of the proposed beamforming scheme via MATLAB simulations. It was assumed that there are 100 sensors randomly located in the coverage area of AP. The sensors were grouped based on the MUI-aware scheduling scheme in Section 3.2. For the sake of illustration, the numbers of blocks along the *x*-direction and *y*-direction were fixed at $B_{x}=B_{y}=5$. One sensor was picked randomly from each group, and the number of total selected sensors was L=20 (five sensor groups were empty). The large-scale fading was modeled using the statistical propagation model for air-terrestrial transmission with low-altitude AP, which was proposed in [14]. In particular, the path loss was modeled as the summation of the free space path loss (in dB) and an extra path loss term (in dB). According to Figure 3 [14], it was assumed that the extra path loss term for each sensor varied randomly from 0 dB to 10 dB. The PAS of the channel was modeled as $S\left(\varphi ,\theta \right)=S_{h}\left(\varphi \right)S_{v}\left(\theta \right)$, where $S_{h}\left(\varphi \right)$ and $S_{v}\left(\theta \right)$ denote the PASs in horizontal and vertical directions, respectively. As in [15,31], $S_{h}\left(\varphi \right)$ and $S_{v}\left(\theta \right)$ were modeled using the von Mises distribution and truncated Laplacian distribution, respectively. The simulation parameters are summarized in Table 1.

**Figure 4:**
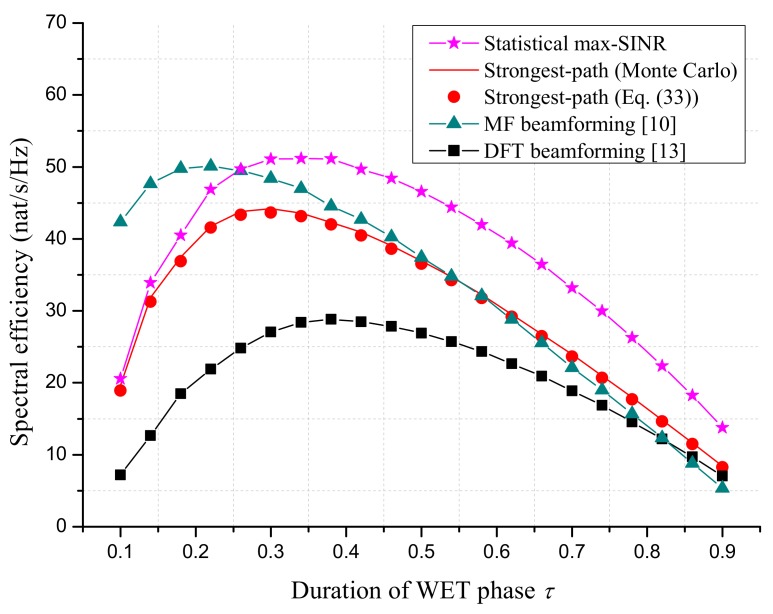
The SE performance versus τ. The number of AP antennas is $N_{x}=N_{y}=25$. The total transmit power of AP is set to $\sum_{l=1}^{L}p_{l}=40$ dBm. The altitude of AP is 100 m. The processing energy of the sensor is set to $E_{l}^{p}=10^{-10}\;J$.

We first compare the SE performance of proposed beamforming schemes with MF beamforming [10] and DFT beamforming [13] in Figure 3 and Figure 4. Note that the MF beamforming required the instantaneous CSI and full number of RF chains (equal to $N_{x}N_{y}$) at AP. The requirement on CSI and the number RF chains for DFT beamforming was the same as the strongest-path beamforming.

Figure 4 simulates the SE performance as a function of the duration of WET phase τ. With the full number of RF chains, it was seen that the proposed statistical max-SINR beamforming achieved a similar performance with MF beamforming at the optimal τ. This makes the statistical max-SINR beamforming attractive, since it did not need the estimation of instantaneous CSI. With the limited number of RF chains, the strongest-path beamforming outperformed the DFT beamforming considerably, since it focused the signal power on the sensor’s direction in a more effective way. When comparing with the MF beamforming, the strongest-path beamforming suffered from an SE loss of 11% due to the lack of instantaneous CSI.

**Figure 5:**
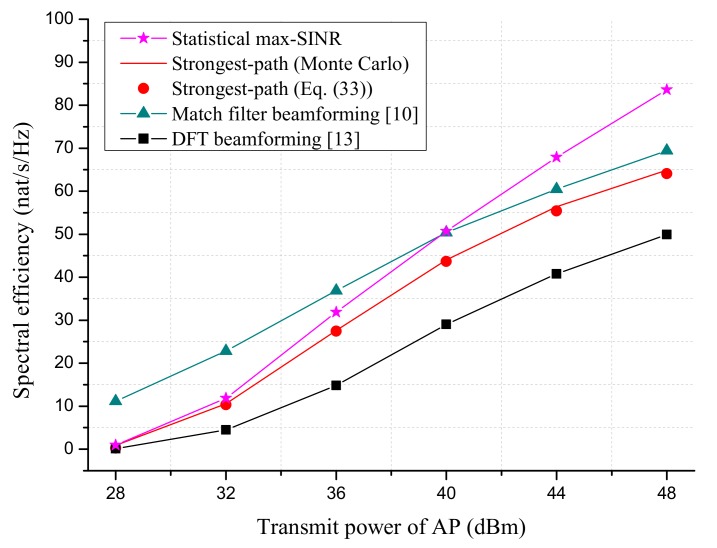
The SE performance versus AP transmit power. The number of AP antennas is $N_{x}=N_{y}=25$. The altitude of AP is 100 m. The processing energy of sensor is set to $E_{l}^{p}=10^{-10}\;J$.

Figure 5 simulates the SE performance for different AP transmit powers, where $\sum_{l=1}^{L}p_{l}$ increases from 28 dBm to 48 dBm. The duration of the WET phase was optimized with respect to SE. It is seen that the SE loss of strongest-path beamforming to the widely-used MF beamforming was quite small (about 6%) for large transmit SNR. However, the SE loss to the statistical max-SINR scheme became larger with the increase of transmit power. This was because the effect of residual MUI was dominant in the large SNR region.

**Figure 6:**
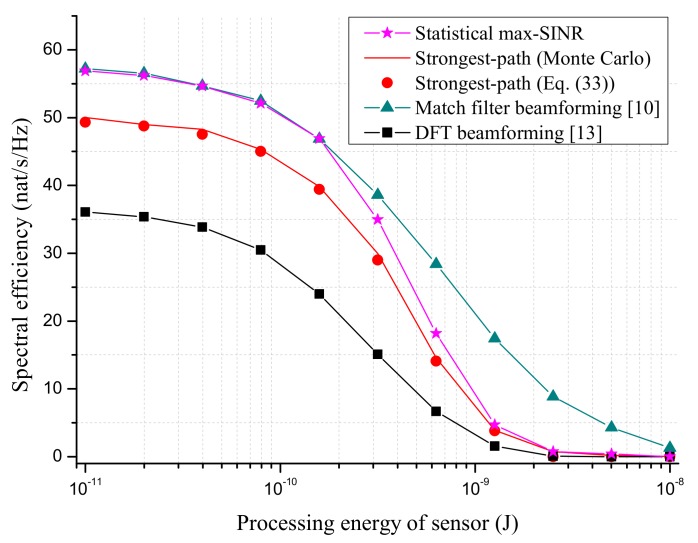
The SE performance versus processing energy of sensor $E_{l}^{p}$. The number of AP antennas is $N_{x}=N_{y}=25$. The total transmit power of AP is set to $\sum_{l=1}^{L}p_{l}=40$ dBm. The altitude of AP is 100 m.

Figure 6 shows the effect of sensor’s processing energy $E_{l}^{p}$ on SE performance, where we assumed that the processing energies for all sensors are the same and vary from $10^{-11}$ J to $10^{-8}$ J, as suggested in [42]. The duration of the WET phase was optimized with respect to SE. As expected, the SEs of all schemes approached zero with the increase of $E_{l}^{p}$ since the energy and time resources left for the WIT phase became less. In this case, AP must increase the transmit power or equip more antennas in order to maintain the system performance. Figure 3 shows the effect of AP’s altitude on the SE performance of proposed beamforming schemes. The duration of the WET phase was optimized to maximize the SE. It is seen that the SE performances of all schemes degraded with the altitude of AP since more path loss was introduced. At last, it is noted from Figure 3 and Figure 4 that the analytical SE based on (33) gave a good approximation to the Monte Carlo result.

**Figure 7:**
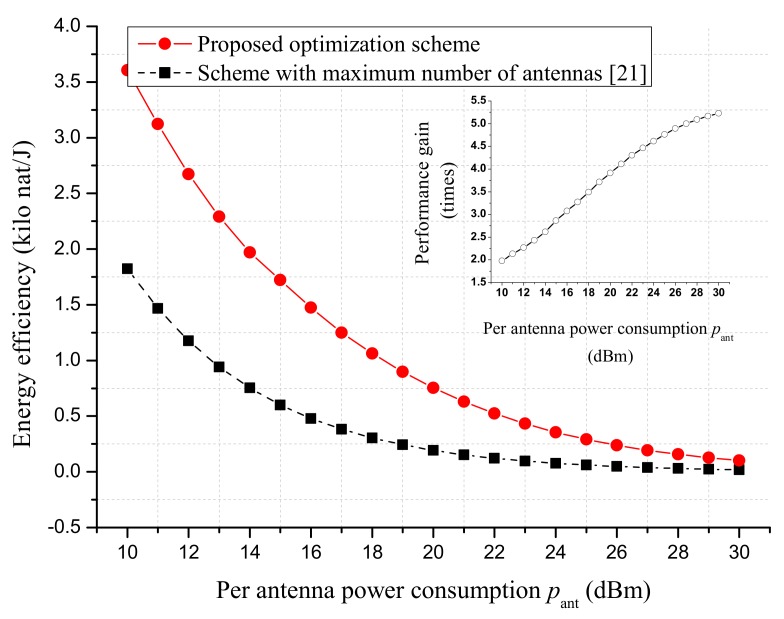
The EE performance versus per antenna circuit power consumption of AP. The maximum number of AP’s antennas is $N_{x}=N_{y}=25$. The altitude of AP is 100 m. The processing energy of sensor is set to $E_{l}^{p}=10^{-10}\;J$. The maximum transmit power of AP is set to $p_{\max}=46$ dBm. The data rate requirement of each sensor is $r_{T}=1$ nat/s/Hz.

Figure 7 shows the EE performance of the strongest-path beamforming as a function of per antenna circuit power consumption $p_{\mathrm{ant}}$. The proposed EE optimization scheme was compared with that in [21] where the maximum number of antennas was used at AP (i.e., ${\tilde{N}}_{x}={\tilde{N}}_{y}=25$). The figure shows that the EE performance was greatly improved by optimizing the number of the AP’s active antennas, especially when the per antenna circuit power consumption $p_{\mathrm{ant}}$ was large. In particular, it is seen that the performance gain increased from 1.9 times to 5.2 times when $p_{\mathrm{ant}}$ varied between 10 dBm and 30 dBm.

**Figure 8:**
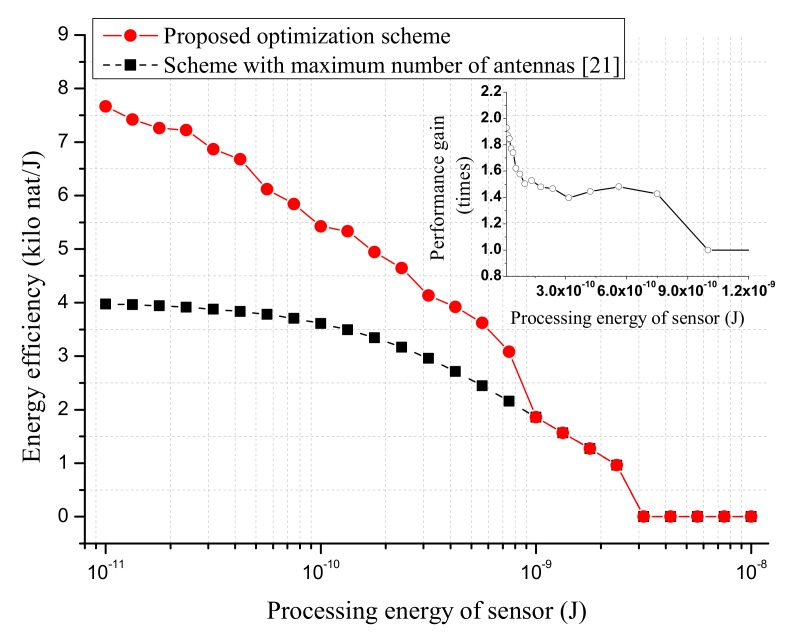
The EE performance versus processing energy of sensor $E_{l}^{p}$. The altitude of AP is 100 m. The per antenna circuit power consumption $p_{\mathrm{ant}}$ is set to 15 dBm. The maximum transmit power of AP is set to $p_{\max}=46$ dBm. The data rate requirement of each sensor is $r_{T}=1$ nat/s/Hz.

**Figure 9:**
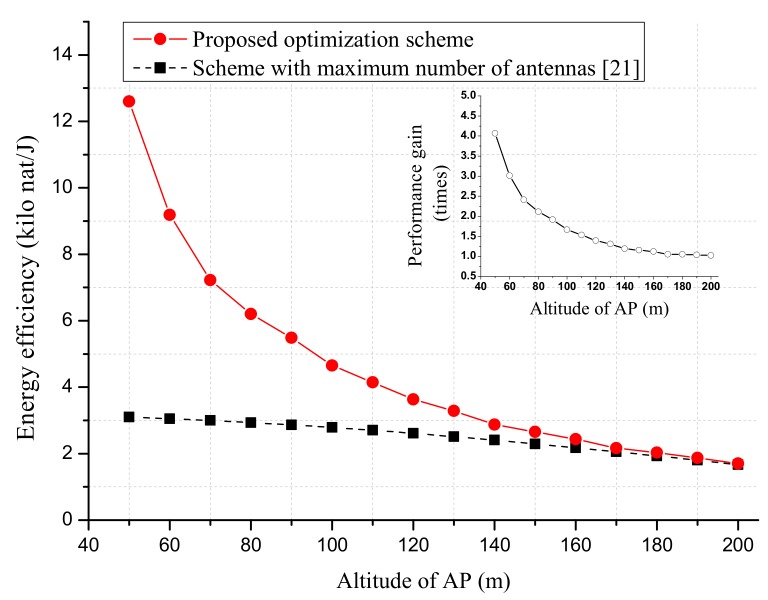
The EE performance versus the altitude of AP. The per antenna circuit power consumption $p_{\mathrm{ant}}$ is set to 20 dBm. The maximum transmit power of AP is set to $p_{\max}=46$ dBm. The data rate requirement of each sensor is $r_{T}=1$ nat/s/Hz.

Figure 8 simulates the effect of processing energy of sensor $E_{l}^{p}$ on the proposed EE optimization scheme. It is seen that, for small $E_{l}^{p}$, the proposed optimization scheme can provide considerable gain. When $E_{l}^{p}$ was greater than $10^{-9}$ J, the EE performance under the proposed optimization scheme converged to that of [21]. This is because in this case, AP must utilize the maximum number of antennas in order to meet the achievable rate constraint and processing energy consumption of the sensor. As a result, the same solution was obtained by the two optimization schemes. Figure 9 shows the effect of AP’s altitude on the proposed EE optimization scheme. Similarly, we can see that the two optimization schemes gave rise to the same EE performance for a large altitude of AP.

## 6. Conclusions

This paper studies the 3D beamforming for an airborne massive MIMO system with WET. When large numbers of RF chains are available at AP, a statistical max-SINR beamforming scheme is proposed to maximize the average received SINR at AP. A heuristic strongest-path beamforming scheme is also proposed when the number of AP RF chains is limited. Under the strongest-path beamforming scheme, the transmit power, number of active antennas of AP and duration of the WET phase are jointly optimized to maximize the system EE. The simulation results show that the statistical max-SINR beamforming scheme can achieve similar SE performance with MF beamforming. The strongest-path beamforming outperforms the DFT beamforming considerably, but suffers from a small SE loss when compared with MF beamforming. Moreover, the proposed EE optimization scheme outperforms the traditional scheme that does not consider the circuit power consumption significantly.

## Figures and Tables

**Table 1:** Simulation parameters.

Parameter	Value
System bandwidth	1 MHz
Thermal noise floor	−100 dBm
Central frequency	1.2 GHz
Rician factor	0 dB
The number of AP antennas	$N_{x}=N_{y}=25$
Angular spread	$\Delta \varphi_{l}=5^{\circ }$, $\Delta \theta_{l}=2.5^{\circ }$
Energy conversion efficiency	η=0.6 [42]
Duration of each frame	T=500 symbol times

## Abbreviations

The following abbreviations are used in this manuscript:

MIMO	Multiple-input multiple-output
AP	Air platform
SINR	Signal-to-interference-plus-noise ratio
WET	Wireless energy transfer
BS	Base station
5G	Fifth generation
EE	Energy efficiency
2D	Two-dimensional
3D	Three-dimensional
WIT	Wireless information transmission
LoS	Line-of-sight
NLoS	Non-LoS
MF	Match filter
RF	Radio frequency
CSI	Channel state information
AoD	Angle of departure
PAS	Power angle spectrum
AWGN	Additive white Gaussian noise
MUI	Multi-user interference
SLNR	Signal-to-leakage-plus-noise ratio
SE	Spectral efficiency
QoS	Quality of service

## Appendix A. Proof of Lemma 1

To prove Lemma 1, we first consider the asymptotic property for dominant eigenvector and eigenvalue of channel covariance matrix $C_{l}$.

According to (5) and the equality (A⊗B)(C⊗D)=(AC)⊗(BD), we have

(A1) $$C_{l}^{\mathrm{LoS}}=\frac{\beta_{l}K_{l}}{K_{l}+1}\left(b\left(\rho_{x,l}\right)⊗a\left(\rho_{y,l}\right)\right){\left(b\left(\rho_{x,l}\right)⊗a\left(\rho_{y,l}\right)\right)}^{H}.$$

From (A1), it is easy to verify that

(A2) $$\begin{array}{c} u_{\max}\left(C_{l}^{\mathrm{LoS}}\right)=\frac{1}{\sqrt{N_{x}N_{y}}}b\left(\rho_{x,l}\right)⊗a\left(\rho_{y,l}\right),\\ \lambda_{\max}\left(C_{l}^{\mathrm{LoS}}\right)=\frac{\beta_{l}K_{l}}{K_{l}+1}N_{x}N_{y}. \end{array}$$

Moreover, using the equality (A⊗B)(C⊗D)=(AC)⊗(BD) on (5) repeatedly, we can obtain

(A3) $$\begin{array}{c} \frac{1}{\sqrt{N_{x}N_{y}}}C_{l}^{\mathrm{NLoS}}b\left({\rho^{\prime }}_{x}\right)⊗a\left({\rho^{\prime }}_{y}\right)\\ =\frac{1}{\sqrt{N_{x}N_{y}}}\frac{\beta_{l}}{K_{l}+1}\int_{\rho_{x,l}^{\min}}^{\rho_{x,l}^{\max}}\int_{\rho_{y,l}^{\min}}^{\rho_{y,l}^{\max}}S_{l}\left(\rho_{x},\rho_{y}\right)b\left(\rho_{x}\right)⊗a\left(\rho_{y}\right)b^{H}\left(\rho_{x}\right)b\left({\rho^{\prime }}_{x}\right)a^{H}\left(\rho_{y}\right)a\left({\rho^{\prime }}_{y}\right)d\rho_{x}d\rho_{y}\\ =\frac{1}{\sqrt{N_{x}N_{y}}}\frac{\beta_{l}}{K_{l}+1}\int_{\rho_{x,l}^{\min}}^{\rho_{x,l}^{\max}}\int_{\rho_{y,l}^{\min}}^{\rho_{y,l}^{\max}}S_{l}\left(\rho_{x},\rho_{y}\right)b\left(\rho_{x}\right)⊗a\left(\rho_{y}\right)\sum_{i=0}^{N_{x}-1}\exp\left(j2\pi i\left({\rho^{\prime }}_{x}-\rho_{x}\right)\right)\sum_{i=0}^{N_{x}-1}\exp\left(j2\pi i\left({\rho^{\prime }}_{y}-\rho_{y}\right)\right)d\rho_{x}d\rho_{y}\\ =\frac{1}{\sqrt{N_{x}N_{y}}}\frac{\beta_{l}}{K_{l}+1}\int_{\rho_{x,l}^{\min}}^{\rho_{x,l}^{\max}}\int_{\rho_{y,l}^{\min}}^{\rho_{y,l}^{\max}}S_{l}\left(\rho_{x},\rho_{y}\right)b\left(\rho_{x}\right)⊗a\left(\rho_{y}\right)\frac{\exp\left(j\pi \left(N_{x}-1\right)\left({\rho^{\prime }}_{x}-\rho_{x}\right)\right)}{\exp\left(j\pi \left(1-N_{y}\right)\left({\rho^{\prime }}_{y}-\rho_{y}\right)\right)}\frac{\sin\left(N_{x}\pi \left({\rho^{\prime }}_{x}-\rho_{x}\right)\right)}{\sin\left(\pi \left({\rho^{\prime }}_{x}-\rho_{x}\right)\right)}\\ \times \frac{\sin\left(N_{y}\pi \left({\rho^{\prime }}_{y}-\rho_{y}\right)\right)}{\sin\left(\pi \left({\rho^{\prime }}_{y}-\rho_{y}\right)\right)}d\rho_{x}d\rho_{y}. \end{array}$$

According to [43], the function $\frac{1}{N}\frac{\sin\left(N\pi x\right)}{\sin\left(\pi x\right)}$ (this function is called aliased sinc function in [43]) converges to standard sinc function sinc(Nx) as N→∞. Therefore, using the limit $\frac{1}{a}\mathrm{sinc}\left(\frac{x}{a}\right)\overset{a\to 0}{\to }\delta \left(x\right)$, we can obtain $\frac{1}{N}\frac{\sin\left(N\pi x\right)}{\sin\left(\pi x\right)}\overset{N\to \infty }{\to }\delta \left(x\right)$. With the above result, in the large $\left\{N_{x},N_{y}\right\}$ limit we can rewrite (A3) as

(A4) $$\begin{array}{c} \frac{1}{\sqrt{N_{x}N_{y}}}C_{l}^{\mathrm{NLoS}}b\left({\rho^{\prime }}_{x}\right)⊗a\left({\rho^{\prime }}_{y}\right)\\ =\frac{1}{\sqrt{N_{x}N_{y}}}\frac{\beta_{l}}{K_{l}+1}S_{l}\left({\rho^{\prime }}_{x},{\rho^{\prime }}_{y}\right)b\left({\rho^{\prime }}_{x}\right)⊗a\left({\rho^{\prime }}_{y}\right). \end{array}$$

Note that in (A4), if ${\rho^{\prime }}_{x}\notin \left[\rho_{x,l}^{\min},\rho_{x,l}^{\max}\right]$ or ${\rho^{\prime }}_{y}\notin \left[\rho_{y,l}^{\min},\rho_{y,l}^{\max}\right]$, we can simply set $S_{l}\left({\rho^{\prime }}_{x},{\rho^{\prime }}_{y}\right)$ to zero. Equation (A4) indicates that $\frac{1}{\sqrt{N_{x}N_{y}}}b\left({\rho^{\prime }}_{x}\right)⊗a\left({\rho^{\prime }}_{y}\right)$ is the eigenvector of $C_{l}^{\mathrm{NLoS}}$, and the corresponding eigenvalue can be expressed as $\frac{\beta_{l}}{K_{l}+1}S_{l}\left({\rho^{\prime }}_{x},{\rho^{\prime }}_{y}\right)$. For the commonly used PAS models, $S_{l}\left(\rho_{x},\rho_{y}\right)$ achieves the maximum at $\left\{\rho_{x},\rho_{y}\right\}=\left\{\rho_{x,l},\rho_{y,l}\right\}$ [15,36], i.e., the virtual AoDs corresponding to sensors’ physical AoDs in horizontal and vertical directions. Therefore, we can conclude

(A5) $$\begin{array}{c} u_{\max}\left(C_{l}^{\mathrm{NLoS}}\right)=\frac{1}{\sqrt{N_{x}N_{y}}}b\left(\rho_{x,l}\right)⊗a\left(\rho_{y,l}\right),\\ \lambda_{\max}\left(C_{l}^{\mathrm{NLoS}}\right)=\frac{\beta_{l}}{K_{l}+1}S_{l}\left(\rho_{x,l},\rho_{y,l}\right). \end{array}$$

Combining (A2) and (A5), in the large $\left\{N_{x},N_{y}\right\}$ limit, we have

(A6) $$\begin{array}{c} u_{\max}\left\{C_{l}\right\}=\frac{1}{\sqrt{N_{x}N_{y}}}b\left(\rho_{x,l}\right)⊗a\left(\rho_{y,l}\right),\\ \lambda_{\max}\left(C_{l}\right)=\frac{\beta_{l}K_{l}}{K_{l}+1}N_{x}N_{y}+\frac{\beta_{l}}{K_{l}+1}S_{l}\left(\rho_{x,l},\rho_{y,l}\right). \end{array}$$

With the channel model in (1) and (2), the harvest energy at sensor *l* can be written as

(A7) $$\begin{array}{cc} P_{l} & =\frac{\eta \tau }{1-\tau }p_{l}w_{E,l}^{H}h_{l}h_{l}^{H}w_{E,l}-\frac{E_{l}^{p}}{\left(1-\tau \right)T}\\ & =\frac{\eta \tau }{1-\tau }p_{l}\left\{w_{E,l}^{H}C_{l}^{\mathrm{LoS}}w_{E,l}+\frac{\beta_{l}}{K_{l}+1}{\left|\int_{\varphi_{l}-\Delta \varphi_{l}}^{\varphi_{l}+\Delta \varphi_{l}}\int_{\theta_{l}-\Delta \theta_{l}}^{\theta_{l}+\Delta \theta_{l}}r_{l}\left(\varphi ,\theta \right)w_{E,l}^{H}b\left(\varphi ,\theta \right)⊗a\left(\varphi ,\theta \right)d\varphi d\theta \right|}^{2}\right\}-\frac{E_{l}^{p}}{\left(1-\tau \right)T} \end{array}$$

Substituting (20) and (A6) into (A7), and using a similar procedure as that in (A3), $P_{l}$ (in the large $\left\{N_{x},N_{y}\right\}$ limit) can be expressed asymptotically as

(A8) $$\begin{array}{cc} P_{l} & =\frac{\eta \tau }{1-\tau }p_{l}\left(\frac{\beta_{l}K_{l}}{K_{l}+1}N_{x}N_{y}+\frac{\beta_{l}}{K_{l}+1}{\left|r_{l}^{v}\left(\rho_{x,l},\rho_{y,l}\right)\right|}^{2}\right)-\frac{E_{l}^{p}}{\left(1-\tau \right)T}, \end{array}$$

where $r_{k}^{v}\left(\rho_{x,k},\rho_{y,k}\right)$ denotes the complex response gain with respect to the virtual AoDs $\left\{\rho_{x},\rho_{y}\right\}$, which can be simply derived by using the formula of integration by substitution. Note that the term $\frac{\beta_{l}}{K_{l}+1}{\left|r_{l}^{v}\left(\rho_{x,l},\rho_{y,l}\right)\right|}^{2}$ becomes negligible in the large $\left\{N_{x},N_{y}\right\}$ limit since $r_{l}^{v}\left(\rho_{x,l},\rho_{y,l}\right)$ is independent with $\left\{N_{x},N_{y}\right\}$. This completes the proof.

## Appendix B. Proof of (31)

According to (5), we have

(A9) $$w_{D,l}^{H}C_{k}w_{D,l}=w_{D,l}^{H}C_{k}^{\mathrm{LoS}}w_{D,l}+w_{D,l}^{H}C_{k}^{\mathrm{NLoS}}w_{D,l},$$

where $w_{D,l}^{H}C_{k}^{\mathrm{LoS}}w_{D,l}$ can be further expanded as

(A10) $$\begin{array}{c} w_{D,l}^{H}C_{k}^{\mathrm{LoS}}w_{D,l}\\ =\frac{1}{N_{x}N_{y}}\frac{\beta_{k}K_{k}}{K_{k}+1}{\left|{\left(b\left(\rho_{x,l}\right)⊗a\left(\rho_{y,l}\right)\right)}^{H}\left(b\left(\rho_{x,k}\right)⊗a\left(\rho_{y,k}\right)\right)\right|}^{2}\\ =\frac{1}{N_{x}N_{y}}\frac{\beta_{k}K_{k}}{K_{k}+1}\frac{\sin^{2}\left(N_{x}\pi \left(\rho_{x,l}-\rho_{x,k}\right)\right)}{\sin^{2}\left(\pi \left(\rho_{x,l}-\rho_{x,k}\right)\right)}\frac{\sin^{2}\left(N_{y}\pi \left(\rho_{y,l}-\rho_{y,k}\right)\right)}{\sin^{2}\left(\pi \left(\rho_{y,l}-\rho_{y,k}\right)\right)}. \end{array}$$

Similarly, $w_{D,l}^{H}C_{k}^{\mathrm{NLoS}}w_{D,l}$ can be expanded as

(A11) $$\begin{array}{c} w_{D,l}^{H}C_{k}^{\mathrm{NLoS}}w_{D,l}=\frac{1}{N_{x}N_{y}}\frac{\beta_{k}}{K_{k}+1}\int_{\rho_{c_{k},x}^{\min}}^{\rho_{c_{k},x}^{\max}}\int_{\rho_{c_{k},y}^{\min}}^{\rho_{ck,y}^{\max}}S_{k}\left(\rho_{x},\rho_{y}\right)\\ \times {\left|{\left(b\left(\rho_{x,l}\right)⊗a\left(\rho_{y,l}\right)\right)}^{H}\left(b\left(\rho_{x}\right)⊗a\left(\rho_{y}\right)\right)\right|}^{2}d\rho_{x}d\rho_{y}\\ =\frac{1}{N_{x}N_{y}}\frac{\beta_{k}}{K_{k}+1}\int_{\rho_{c_{k},x}^{\min}}^{\rho_{c_{k},x}^{\max}}\int_{\rho_{c_{k},y}^{\min}}^{\rho_{ck,y}^{\max}}S_{k}\left(\rho_{x},\rho_{y}\right)\prod_{i\in \left\{x,y\right\}}\frac{\sin^{2}\left(N_{x}\pi \left(\rho_{i,l}-\rho_{i}\right)\right)}{\sin^{2}\left(\pi \left(\rho_{i,l}-\rho_{i}\right)\right)}d\rho_{y}. \end{array}$$

Inserting (A9)–(A11) into (30), we obtain (31).

## Appendix C. Proof of Lemma 2

We first prove the first part of the lemma. Without loss of generality, we assume that only condition (a) is satisfied. From (31), the power of MUI can be rewritten as

(A12) $$\begin{array}{cc} Q_{l}^{\mathrm{MUI}} & =\sum_{k=1,k\neq l}^{L}\;\;\;\;P_{k}w_{D,l}^{H}C_{k}^{\mathrm{LoS}}w_{D,l}+\;\;\sum_{k=1,k\neq l}^{L}\;\;\;\;P_{k}w_{D,l}^{H}C_{k}^{\mathrm{NLoS}}w_{D,l}\\ & =Q_{l,\mathrm{LoS}}^{\mathrm{MUI}}+Q_{l,\mathrm{NLoS}}^{\mathrm{MUI}}. \end{array}$$

The first and second terms of right-hand side represent the MUIs contributed by the LoS and NLoS channels, respectively. By using the expression of $P_{k}$ in Lemma 1 and (A10), we can obtain (in the large $\left\{N_{x},N_{y}\right\}$ limit)

(A13) $$\begin{array}{cc} \frac{Q_{l,\mathrm{LoS}}^{\mathrm{MUI}}}{Q_{l}^{U}} & \leq \frac{1}{N_{x}N_{y}}\sum_{k=1,k\neq l}^{L}\frac{P_{k}}{P_{l}}\frac{\sin^{2}\left(N_{x}\pi \left(\rho_{x,l}-\rho_{x,k}\right)\right)}{N_{x}\sin^{2}\left(\pi \left(\rho_{x,l}-\rho_{x,k}\right)\right)}\frac{\sin^{2}\left(N_{y}\pi \left(\rho_{y,l}-\rho_{y,k}\right)\right)}{N_{y}\sin^{2}\left(\pi \left(\rho_{y,l}-\rho_{y,k}\right)\right)}. \end{array}$$

Note that the function $\frac{1}{N}\frac{\sin\left(N\pi x\right)}{\sin\left(\pi x\right)}$ converges to standard sinc function sinc(Nx) as N→∞ [43]. Thus, when condition (a) is satisfied, i.e., $\left[\rho_{x,l}^{\min},\rho_{x,l}^{\max}\right]\cap \left[\rho_{x,k}^{\min},\rho_{x,k}^{\max}\right]=\emptyset$, ∀l≠k, we can obtain

(A14) $$\begin{array}{cc} \frac{\sin^{2}\left(N_{x}\pi \left(\rho_{x,l}-\rho_{x,k}\right)\right)}{N_{x}\sin^{2}\left(\pi \left(\rho_{x,l}-\rho_{x,k}\right)\right)} & =N_{x}\frac{\sin^{2}\left(N_{x}\pi \left(\rho_{x,l}-\rho_{x,k}\right)\right)}{N_{x}^{2}\pi^{2}{\left(\rho_{x,l}-\rho_{x,k}\right)}^{2}}\\ & \leq \frac{1}{N_{x}\pi^{2}{\left(\rho_{x,l}-\rho_{x,k}\right)}^{2}}=O\left(N_{x}^{-1}\right), \end{array}$$

where the second step follows from the inequality |sinx|≤1. Moreover, the function $\frac{1}{N}\frac{\sin\left(N\pi x\right)}{\sin\left(\pi x\right)}$ achieves its maximum at point x=0, thus, we have

(A15) $$\frac{\sin^{2}\left(N_{y}\pi \left(\rho_{y,l}-\rho_{y,k}\right)\right)}{N_{y}\sin^{2}\left(\pi \left(\rho_{y,l}-\rho_{y,k}\right)\right)}\leq N_{y}.$$

Substituting (A14) and (A15) into (A13), we can obtain $\frac{Q_{l,\mathrm{LoS}}^{\mathrm{MUI}}}{Q_{l}^{U}}\leq O\left(N_{x}^{-2}\right)$. Using a similar procedure as that in the appendix of [44], we can prove $\frac{Q_{l,\mathrm{NLoS}}^{\mathrm{MUI}}}{Q_{l}^{U}}\leq O\left(N_{x}^{-2}\right)$. This completes the proof for the first part of Lemma 2.

When both conditions (a) and (b) are satisfied, the proof is similar with that in appendix of [44]. Thus, it is omitted.
